# Wiskott-Aldrich Syndrome: A Report of a Rare X-Linked Disorder

**DOI:** 10.7759/cureus.67658

**Published:** 2024-08-24

**Authors:** Manojkumar G Patil, Sarita Verma, Om Prasanth Reddy Avuthu, Kannan Subramanian, Sampada Tambolkar, Shailaja V Mane

**Affiliations:** 1 Pediatrics, Dr. D. Y. Patil Medical College, Hospital & Research Centre, Dr. D. Y. Patil Vidyapeeth (Deemed to be University), Pune, IND; 2 Pediatrics, King Edward Memorial Hospital, Pune, IND; 3 Hematology, King Edward Memorial Hospital, Pune, IND

**Keywords:** whole-exome sequencing, x-linked inheritance, hematopoietic stem cell transplant, clinical hematology, pediatrics & neonatology

## Abstract

Wiskott-Aldrich syndrome (WAS) is a rare X-linked recessive genetic disorder marked by eczema, thrombocytopenia, and immunodeficiency. The associated immune dysregulation increases the risk of autoimmune disorders and lymphoid malignancies. WAS results from mutations in the WAS protein gene on the short arm of the X chromosome. Here, we present the case of a seven-month-old male, born to non-consanguineous parents with no significant birth or family history. The child had height, weight, and head circumference below the third percentile for age and presented with recurrent mild upper respiratory infections, mild eczema, and thrombocytopenia. Despite symptomatic treatment and clinical improvement, platelet counts continued to decline. A provisional diagnosis of immune thrombocytopenia was made, and intravenous immunoglobulin was administered, which halted the downward trend but did not improve platelet counts. Autoimmune testing revealed strong positivity for antinuclear antibodies (ANA). Given the early-onset thrombocytopenia, anemia, and failure to thrive, autoimmune lymphoproliferative syndrome was suspected. However, T cell subset analysis was normal. A bone marrow biopsy suggested myelodysplastic syndrome or myeloproliferative neoplasm, but molecular studies were negative. Due to the early-onset autoimmunity and strongly positive ANA, genetic testing via whole exome sequencing confirmed the diagnosis of WAS.

## Introduction

Wiskott-Aldrich syndrome (WAS) is a rare X-linked recessive genetic disorder with an incidence of approximately 1 in 100,000 live births [1]. First described by Alfred Wiskott in 1937 and later by Robert A. Aldrich in 1954 [2], WAS is characterized by a triad of eczema, thrombocytopenia leading to bleeding manifestations, and recurrent infections due to immunodeficiency. The disorder also predisposes individuals to autoimmune disorders and lymphoid malignancies due to immune dysregulation and can result in failure to thrive.

WAS is caused by mutations in the WAS Protein (WASP) gene located on the short arm of the X chromosome. This gene comprises 12 exons and encodes a 502-amino-acid protein essential for immune cell function. Mutations in the WASP gene disrupt immune responses and platelet production. WASP is expressed in hematopoietic, myeloid, and lymphoid stem cells, playing a crucial role in regulating the immunological synapse between T and B cells, as well as in cytoskeletal remodeling and actin polymerization.

The clinical presentation of WAS can vary widely, from the severe classical form to milder variants such as X-linked neutropenia (XLN) and X-linked thrombocytopenia (XLT) [3,4]. Diagnosis typically occurs around 12 months of age, although cases have been identified as early as 5.5 months [5].

We present the case of a seven-month-old boy with recurrent upper respiratory infections and thrombocytopenia unresponsive to intravenous immunoglobulin (IVIG). Early-onset autoimmunity was indicated by strong antinuclear antibody (ANA) positivity.

## Case presentation

A seven-month-old boy, the firstborn in a non-consanguineous marriage, presented with a cough, cold, and fever for two days, accompanied by a mild exanthematous maculopapular rash on his upper limbs, abdomen, and lower limbs. His weight, length, and head circumference were 6 kg, 68 cm, and 40 cm, respectively, all below the third percentile for his age. On examination, the child appeared mildly irritable but was feeding well and was hemodynamically stable. The respiratory system examination revealed no abnormal findings. He had an uneventful neonatal period, and the mother’s antenatal history was unremarkable, with no similar complaints in the family. Over the previous two months, he had experienced two similar episodes of upper respiratory tract infections and had been managed symptomatically with antipyretics on an outpatient basis. Due to persistent fever on the fourth day, a complete blood count and urine microscopy were performed. Urine microscopy was normal, and the initial hemogram showed hemoglobin of 7.5 g/dl with indices suggestive of iron deficiency anemia, thrombocytopenia of 89,000/µL, and mild leukocytosis (Table 1).

**Table 1:** Initial hemogram of the child

Investigation	Reference value	Patient value
Hemoglobin	11-14.5 g/dL	7.5 g/dL
Total leukocyte count	4,000-12,000/μL	14,300/μL
Neutrophils	40-80%	68%
Lymphocytes	20-40%	24%
Monocytes	2-10%	4%
Eosinophils	1-6%	4%
Basophils	1-2%	0%
Platelet count	150,000-410,000/μL	89,000/μL
Packed cell volume	33-43%	23.10%
Mean corpuscular volume	74-89 fL	67.6 fL
Mean corpuscular hemoglobulin	24-30 pgms	21.90 pgms
Mean corpuscular hemoglobulin concentration	24-30 pgms	32.40 g/dL
Mean platelet volume	7.4-11.4 fL	7.2 fL

Although the child improved symptomatically with supportive medication, a subsequent hemogram taken three days later revealed a decreasing platelet count, which had dropped to 50,000/µL. The child was monitored on an outpatient basis, with follow-up every alternate day. After 10 days without fever, the platelet count fell to 18,000/µL, prompting admission for further evaluation, including bone marrow aspiration and biopsy. A provisional diagnosis of immune thrombocytopenia was made, and IVIG was administered at a dose of 1 g/kg. The platelet count stopped decreasing after 24 hours but did not increase. After 72 hours, the platelet count stabilized at 27,000/µL without further improvement. Given the early-onset thrombocytopenia, anemia, and failure to thrive, autoimmune lymphoproliferative syndrome was also suspected. T cell subset analysis was performed and returned normal (Table 2).

**Table 2:** Lymphocyte subset analysis

Investigation	Reference value	Patient’s reports
Total leucocyte count	6,000-18,000 mm^3^	14,050 mm^3^
Lymphocytes	41-71%	39.60%
Absolute lymphocyte count	4,000-10,000 mm^3^	5,570 mm^3^
Absolute CD19 cell count	610-2,600 mm^3^	639 mm^3^
CD19 percentage	14-37%	13.20%
Absolute CD3 count	1,900-5,900 mm^3^	1,815 mm^3^
CD3 percentage	49-76%	37%
Absolute CD8 count	500-1,700 mm^3^	245 mm^3^
Absolute CD4 count	1,400-4,300 mm^3^	1,489 mm^3^

**Figure 1:**
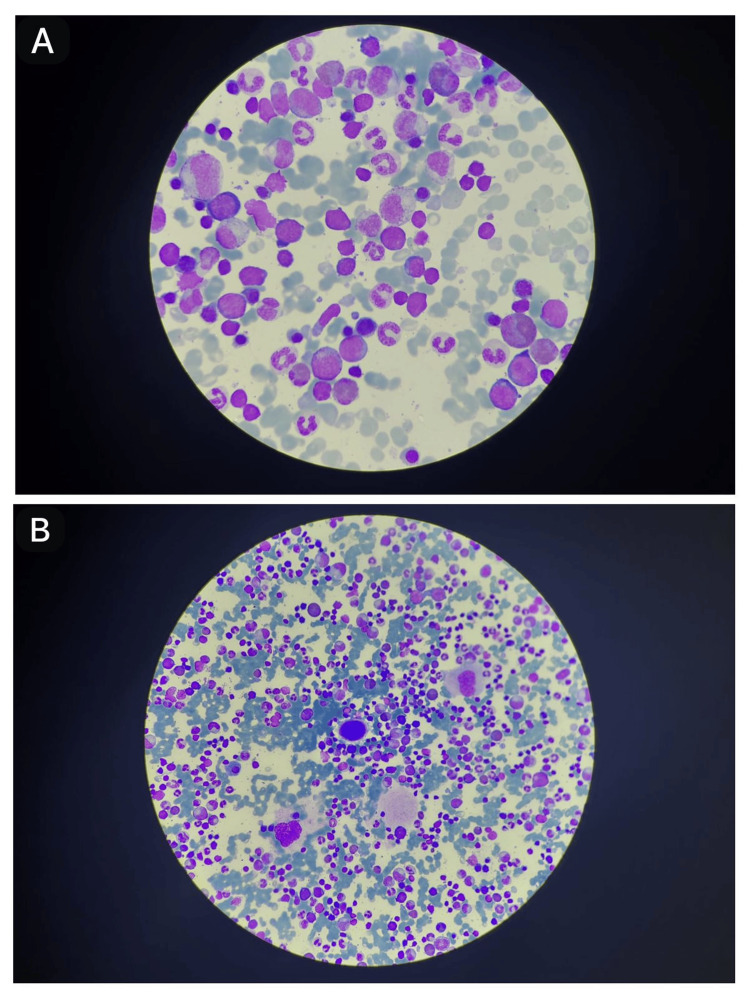
Photomicrograph of bone marrow aspirate showing (A) cells with blast-like morphology along with myeloid hyperplasia and (B) adequate megakaryocytes

Autoimmune studies revealed a strongly positive ANA with a titer of 1:1,000 and the presence of a speckled pattern. The final bone marrow biopsy indicated mild myeloid hyperplasia, increased eosinophils and blasts, and adequate megakaryocytes, suggestive of myelodysplastic syndrome and myeloproliferative neoplasm. However, subsequent molecular testing returned negative results (Figure 1).

The early onset of autoimmunity, indicated by a strongly positive ANA and a skin rash, raised suspicion of primary immunodeficiency, leading to genetic testing. To stabilize the platelet count while awaiting results, we administered weekly injections of romiplostim. Two doses improved the platelet count to a maximum of 75,000/µL, resulting in the child’s discharge after a 10-day hospital stay. Whole exome sequencing revealed the gene variant c.756G>A (p.Trp252Ter) consistent with WAS. The child is on monthly follow-up and currently does not exhibit bleeding manifestations or recurrent severe infections, although there remains potential for these symptoms to develop in the future.

## Discussion

WAS is an X-linked disorder that predominantly affects males, with females typically being carriers. Affected females are rare due to normal X chromosome inactivation [6,7]. Thrombocytopenia in WAS arises from defective platelet production and increased phagocytosis by the reticuloendothelial system, sometimes improving post-splenectomy. Eczema, often severe, may result from dendritic cell dysfunction and imbalanced TH2 immunity. Patients with WAS are also prone to allergic rhinitis, asthma, and food allergies. The condition compromises innate immunity, leading to reduced chemotaxis and phagocytosis in monocytes and macrophages, defective neutrophil adhesion, and impaired dendritic cell and NK cell function. Adaptive immunity is also affected, increasing susceptibility to intracellular pathogens. Autoimmune disorders are prevalent due to the failure of regulatory T cells to suppress autologous CD4+ effector T cells. WAS patients are at higher risk for malignancies, particularly non-Hodgkin lymphomas at extranodal sites, lymphoblastic leukemia, myelodysplasia, and myeloproliferative disorders, due to impaired immune surveillance [8].

A multicenter study by Suri et al. analyzed 95 WAS patients: 81 with classic WAS and 14 with XLT. The mean age at presentation for classic WAS was three months and diagnosis at 12 months, while for XLT, it was six and 51 months, respectively. Bleeding was the most common presenting symptom (74%), mainly gastrointestinal, followed by eczema (46.3%) and infections (43%), notably pneumonia and otitis media. The clinical triad was initially seen in 15% of cases but developed in 73% over time. Autoimmune manifestations were present in 3% of cases at presentation, with over 40% developing them later; 9% tested positive for ANA [5]. Our case presented with thrombocytopenia, mild eczema, and mild infections appropriate for age, with early-onset autoimmunity indicated by a strongly positive ANA, prompting genetic testing.

The diagnosis of WAS in male patients should involve evaluating clinical history, family history, physical examination findings, and laboratory data. Mutations causing absent WASP expression lead to classic WAS, while residual expression results in XLT, and gain-of-function mutations lead to XLN. Flow cytometry analysis of WASP expression is a recommended initial screening tool, indicating disease, carrier status, or mixed chimerism post-transplantation. If WASP expression is normal, further testing should be based on clinical suspicion. A confirmatory diagnosis requires identifying WASP gene mutations. Over 300 mutations have been identified, with a study by Suri et al. analyzing 67 cases and finding 47 variants. Nonsense and missense mutations were the most common, with frameshift and nonsense mutations causing premature protein termination being prominent. WAS patients showed a higher prevalence of nonsense mutations, while missense mutations were more common in XLT patients [5]. Our case identified the gene variant c.756G>A (p.Trp252Ter) in exon 8, classified as likely pathogenic, leading to premature protein termination [9].

The clinical scoring system for WAS evaluates five parameters: thrombocytopenia, eczema severity, infections, autoimmunity, and malignancy, each scoring 1 point for a total of 5. A score of 3-4 indicates a severe phenotype, while a score of 5 includes autoimmunity or malignancy. Scores of 3 or higher suggest a severe phenotype, while scores below 3 indicate a milder phenotype, or XLT, characterized by thrombocytopenia with mild or no eczema and infrequent infections. Our patient had a score of 3 at discharge [10].

Preventing complications from recurrent infections in WAS patients is crucial for reducing mortality and morbidity. IVIG is used as a supportive treatment for infections and autoimmune complications but does not improve platelet counts. Splenectomy may improve platelet counts but is not recommended if hematopoietic stem cell transplantation (HSCT) is planned due to increased mortality risk [10].

Allogeneic HSCT is the primary treatment for WAS, providing long-term improvement in symptoms related to immunodeficiency and thrombocytopenia. Since its implementation in 1968, HSCT outcomes have significantly improved [11]. Gene therapy involving the transplantation of autologous, gene-corrected hematopoietic stem cells has shown promise. A study by Hacein-Bey Abina et al. using a lentiviral vector demonstrated improvements in thrombocytopenia, eczema, susceptibility to infections, and autoimmune manifestations in most patients [12].

## Conclusions

This case involves a seven-month-old male who presented with recurrent upper respiratory infections, thrombocytopenia, failure to thrive, and early-onset autoimmunity, underscoring the importance of early diagnosis and intervention in WAS. Early recognition and genetic testing are crucial for confirming the diagnosis and guiding appropriate treatment strategies to prevent infections and autoimmune complications, thereby improving the long-term prognosis. Timely diagnosis also facilitates planning for HSCT or gene therapy, which can offer potential cures and enhance both quality of life and patient outcomes.
